# Site-specific antigen-adjuvant conjugation using cell-free protein synthesis enhances antigen presentation and CD8^+^ T-cell response

**DOI:** 10.1038/s41598-021-85709-1

**Published:** 2021-03-18

**Authors:** Adam M. Weiss, Jainu Ajit, Tyler J. Albin, Neeraj Kapoor, Shilpa Maroju, Aym Berges, Lucy Pill, Jeff Fairman, Aaron P. Esser-Kahn

**Affiliations:** 1 Pritzker School of Molecular Engineering, University of Chicago, 5640 S. Ellis Ave, Chicago, IL 60637 USA; 2 Department of Chemistry, University of Chicago, 5735 S Ellis Ave., Chicago, IL 60637 USA; 3 Department of Chemistry, University of California, 1102 Natural Sciences 2, Irvine, CA 92617 USA; 4 Vaxcyte, Inc., 353 Hatch Drive, Foster City, CA 94404 USA

**Keywords:** Vaccines, Immunochemistry, Synthetic biology, Protein design, Molecular engineering, Pattern recognition receptors, Antigen processing and presentation

## Abstract

Antigen-adjuvant conjugation is known to enhance antigen-specific T-cell production in vaccine models, but scalable methods are required to generate site-specific conjugation for clinical translation of this technique. We report the use of the cell-free protein synthesis (CFPS) platform as a rapid method to produce large quantities (> 100 mg/L) of a model antigen, ovalbumin (OVA), with site-specific incorporation of *p*-azidomethyl-l-phenylalanine (pAMF) at two solvent-exposed sites away from immunodominant epitopes. Using copper-free click chemistry, we conjugated CpG oligodeoxynucleotide toll-like receptor 9 (TLR9) agonists to the pAMF sites on the mutant OVA protein. The OVA-CpG conjugates demonstrate enhanced antigen presentation in vitro and increased antigen-specific CD8^+^ T-cell production in vivo. Moreover, OVA-CpG conjugation reduced the dose of CpG needed to invoke antigen-specific T-cell production tenfold. These results highlight how site-specific conjugation and CFPS technology can be implemented to produce large quantities of covalently-linked antigen-adjuvant conjugates for use in clinical vaccines.

## Introduction

Synthetic subunit vaccines that invoke potent cellular immune responses are desirable for the safe and scalable prevention of disease. Early vaccines were composed of whole pathogens attenuated by heat inactivation or chemical modification. Traditional attenuated vaccines are highly potent, and their administration has led to reductions in morbidity from many diseases^1^. Despite their potency, attenuated vaccines pose a safety risk resulting from the presence of live pathogens, limiting use in elderly or immunocompromised individuals. Furthermore, attenuated vaccines can contain harmful pathogenic material, undergo spontaneous mutations to revert to their infectious form, and risk infecting the host if incompletely inactivated^2^. Thus, there is interest in designing synthetic alternatives to attenuated vaccines.

Subunit vaccines comprising a protein antigen do not contain live pathogenic material and therefore serve as desirable alternatives to attenuated vaccines. Subunit vaccines are composed of poorly immunogenic protein antigens co-administered with one or more adjuvants which bind pattern-recognition receptors (PRRs) to activate innate immunity^2–5^. Innate immune activation is critical for cross-presentation of proteolyzed antigen on MHC by antigen-presenting cells (APCs) and recognition by antigen-specific T-cells, resulting in protective adaptive immune responses^4^. FDA-approved subunit vaccines containing synthetic adjuvants have been developed for hepatitis B and shingles, among others^6,7^.

Despite the success of subunit vaccines, they have been limited by weak immunogenicity of the antigen, necessitating co-administration of large doses of adjuvant. Synthetic PRR agonists often generate adverse inflammatory profiles when used as adjuvants, resulting in poor translation to clinical use^8^. One method to reduce the dose of agonist is through co-delivery of antigen and adjuvant to the same APC^9^. Co-delivery allows adjuvant-mediated activation of the APC along with concurrent antigen processing and presentation, resulting in efficient T-cell responses and dose sparing^10^. Precise control over the amount of adjuvant administered with the antigen can prevent adverse responses and improve efficiency of subunit vaccines.

Co-delivery of antigen and adjuvant through conjugation can facilitate efficient activation of APCs and enhanced antigen exposure using a single construct, enhancing proliferation of antigen-specific T-cells^9,14,15^. Oligodeoxynucleotides, such as unmethylated CpG, activate the innate immune system by binding endosomal toll-like receptor 9 (TLR9) to enhance cross-presentation of antigenic components^16^. TLR9 agonists conjugated to ovalbumin (OVA) were shown to enhance both cross-presentation and antigen-specific CD8^+^ T-cell production^9,11^. However, these systems were limited by poor control over the modification site and aggregation resulting from over-conjugation of ODN adjuvants. To overcome this limitation, we hypothesized that cell-free protein synthesis would allow scalable production of dose controlled, site-specific antigen-adjuvant conjugates that would not affect protein folding or disrupt major epitopes.

Cell-free protein synthesis (CFPS) is an efficient, *E.coli*-derived platform to express and purify unglycosylated proteins containing non-native amino acids (nnAA) in their natively-folded state. The translational machinery provided by the cellular lysate supplemented with energy sources and plasmid DNA facilitates in vitro synthesis of proteins that are unnatural, insoluble, or toxic to living systems^17^. Historically, CFPS systems were limited by feedback inhibition caused by byproducts resulting from use of ATP as an energy source^18^. Recently, the XpressCF^+^ CFPS system (Vaxcyte, Inc.) has overcome these limitations by using pyruvate as an alternative energy source to allow scalable production of complex proteins^18,19^. By using an orthogonal tRNA and aminoacyl synthetase pairing, this technology allows site-specific incorporation of nnAAs with reactive side chains into the folded protein^19–21^. Using this system, we aimed to test if scalable production of site-specific antigen-adjuvant conjugates could induce production of a CD8^+^ killer T cell response in a vaccination model.

Herein, we describe a novel strategy to generate a model antigen-adjuvant conjugate subunit vaccine using CFPS. OVA-2pAMF, an unglycosylated OVA mutant containing two site-specific *p*-azidomethyl-L-phenylalanine (pAMF) mutations at K20 and K370, was synthesized using XpressCF^+^ on a 14 mg scale. A TLR9 agonist, CpG1018, was conjugated to the pAMF sites using azide-alkyne click chemistry to generate the conjugate vaccine, OVA-CpG. Functionally, the conjugate vaccine enhanced antigen cross-presentation and APC activation in vitro and promoted CD8^+^ T-cell responses in vivo without showing aggregation or cytotoxicity. Most importantly, these studies demonstrate that large quantities of mutant proteins containing nnAAs at specific sites in the folded protein can be prepared using CFPS and that conjugating a CpG oligonucleotide TLR agonist to these sites can enhance antigen-specific CD8^+^ T-cell production in a vaccination model.

## Results

### Synthesis of OVA-CpG conjugates using CFPS

CFPS is a versatile approach to synthesize protein antigens with site-specific mutations for antigen-adjuvant conjugation. To demonstrate the applicability of CFPS and to provide an appropriate comparison to previous antigen-adjuvant co-localization experiments^9–15^, we used a model antigen, OVA. Noting that a pioneering study of non-specific conjugation of CpG to OVA at a ≥ 2:1 ratio induced aggregation and reduced antigen presentation^11^, we introduced two site-specific conjugatable handles on one OVA protein to overcome this limitation of non-specific conjugation. We selected the human-mouse cross-reactive CpG1018 as a clinically relevant TLR9 agonist and appended a commercially available 5′-thiol handle for conjugation^6^.

**Figure 1:**
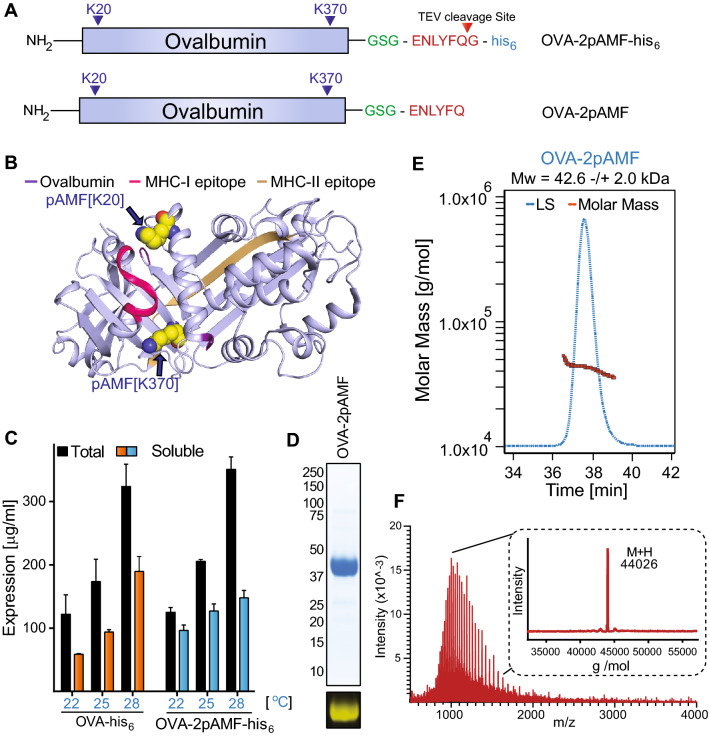
Expression, purification, and characterization of OVA-2pAMF. (**A**) Modular architecture and expression construct design of OVA-2pAMF with (**B**) the K20 and K370 pAMF conjugation sites noted. (**C**) Optimization of OVA-2pAMF expression conditions. (**D**) SDS-PAGE demonstrates > 95% purity, and DBCO-TAMRA labeling confirms pAMF incorporation. Biophysical characterization of purified OVA-2pAMF using (**E**) SEC-MALS indicates a monodisperse protein preparation and F) Q-TOF ESI–MS (deconvoluted in inset) indicates close agreement with the expected mass, 44,026 Da.

In selecting the conjugation sites, we considered the structure of OVA with a focus on its immunodominant MHC epitopes, SIINFEKL (OVA_257-264_) and ISQAVHAAHAEINEAGR (OVA_323-339_). We hypothesized that epitope blocking and aggregation could be mitigated by introducing spatially-isolated and solvent-exposed conjugation sites away from the immunodominant epitopes. K20 and K370 were therefore selected as optimal conjugation sites (Fig. 1A,B). The handles were introduced by replacing lysine codons with amber codons in the coding sequence for OVA. Using an orthogonal tRNA and synthetase pair, we selectively incorporated *p*-azidomethyl-l-phenylalanine (pAMF) at these sites to generate one protein with two azide reactive sites, OVA-2pAMF. A TEV protease cleavage site coupled to a poly-Histidine tag was expressed at the C terminus to facilitate purification of the protein by sequential combination of affinity and size-exclusion chromatography. Protein expression conditions were optimized to a temperature of 25 °C (Fig. 1C) and the purity of the purified protein was confirmed by SDS-PAGE (Figs. 1D and S1). The protein was further characterized by Q-TOF MS and MALS (Fig. 1D–F). CFPS was subsequently scaled up to generate 14 mg of the purified protein. After purification, endotoxin was removed using Triton X-114 to a level < 1.5 EU/mL^22^. A recovery yield of 78% for the endotoxin purification step was achieved (Fig. S2), permitting use of milligram-scale OVA-2pAMF in later biological experiments.

**Figure 2:**
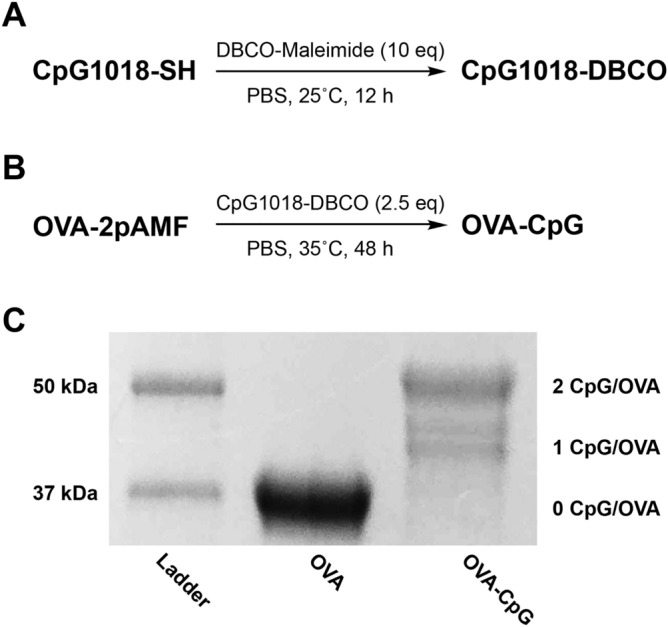
(**A**) Synthesis of CpG-DBCO and (**B**) conjugation of CpG-DBCO to OVA-2pAMF. (**C**) SDS-PAGE gel of OVA-CpG conjugates visualized using One-Step Blue protein gel staining demonstrates loading of 1.5 OVA/CpG in batch one of the OVA-CpG conjugate.

After synthesizing and purifying OVA-2pAMF, we conjugated the 5′-thiol modified CpG1018 through a DBCO-Maleimide linker (Figs. 2A,B and S3). Copper-free click chemistry was employed to facilitate this reaction under aqueous conditions. Unreacted CpG-DBCO was removed by centrifugal filtration, and the OVA-CpG conjugates were characterized using SDS-PAGE gel chromatography. Three batches of OVA-CpG were prepared, and loading densities of 1.2, 1.5, and 1.3 CpGs/OVA were obtained (Figs. 2C and S4). It should be noted that SDS-PAGE revealed two distinct peaks for the 1 CpG/OVA component; while both components were included in the densitometry analysis, we believe these species correspond to different charge states of the K20 or K370 conjugation sites after reaction with CpG which may have resulted in minor electrostatic variations during electrophoresis. We also attempted to improve loading by increasing reaction temperature, but these efforts resulted in degradation of OVA-2pAMF observed through the presence of large, low molecular weight bands ≤ 37 kDa in SDS-PAGE gels of the crude product (not shown). Addition of excess CpG-DBCO or prolonging the reaction were also found to be insufficient to drive the reaction to completion; we believe that the negative charge of CpG could shield the pAMF sites during later stages of the reaction to result in this effect. Unreacted CpG was found to be sufficiently removed using size-exclusion HPLC of the purified product (Fig. S5). Preparative anion exchange chromatography using a 0–1 M NaCl gradient in a method previously described by the Chertok group was shown to remove unreacted OVA-2pAMF from the reaction mixture; however, components containing one and two CpG/OVA eluted together (Fig. S6)^11^. With the limited benefit to this purification strategy, we opted to use the unpurified heterogeneous mixtures in future studies; thus, site-specific OVA-CpG conjugates containing 1.2–1.5 CpG/OVA were achieved for use in subsequent experimentation.

### In vitro immunostimulatory activity of conjugates

**Figure 3:**
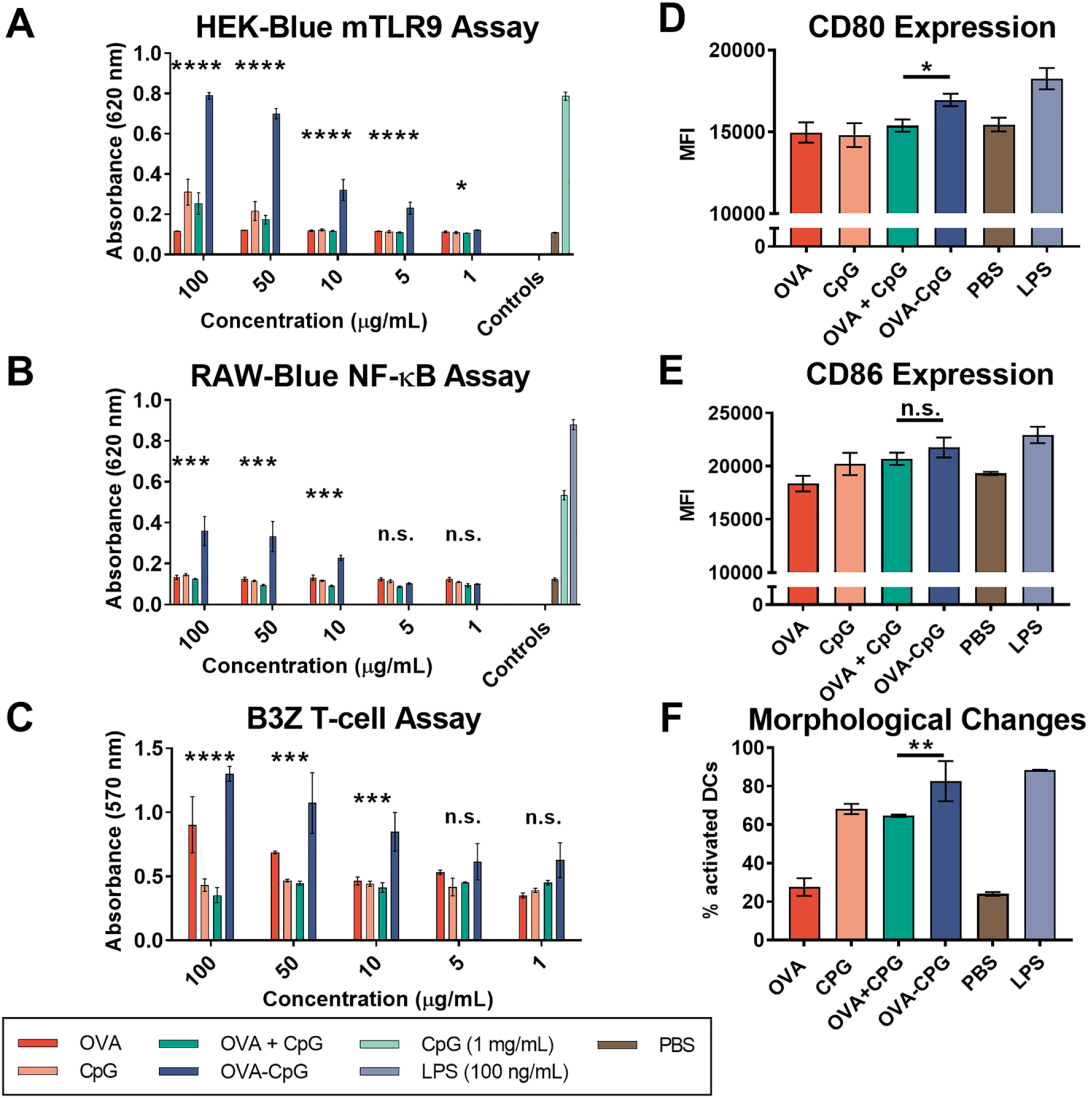
In vitro TLR9 activity of OVA-CpG conjugates in (**A**) HEK mTLR9 and (**B**) RAW-Blue NF-κB reporter cell activity was enhanced in OVA-CpG conjugates relative to unlinked controls. (**C**) Cross-presentation of OVA-CpG on DC2.4 cells was enhanced relative to unlinked controls as measured using the B3Z T-cell hybridoma model. Flow cytometry evaluating mean fluorescent intensity of (**D**) CD80 and (**E**) CD86 as well as (**F**) morphological changes associated with activation after stimulation with 50 μg/mL OVA-CpG or unlinked controls. Statistics were conducted using one- or two-way ANOVA with multiple comparisons testing to evaluate OVA-CpG relative to OVA + CpG.

Having successfully synthesized OVA-CpG, we validated its in vitro immune activity relative to unlinked controls using RAW-Blue and HEK mTLR9 reporter cell lines. Conjugation of PRR agonists to a macromolecule can alter receptor binding, and it is therefore important to validate activity after conjugation. We first tested activity using HEK mTLR9 reporter cells. OVA-CpG conjugates exhibited enhanced mTLR9 activation relative to unlinked OVA + CpG (Fig. 3A). For all in vitro studies, the dose of OVA and CpG used in the OVA + CpG treatment was designed to match that of the linked OVA-CpG conjugate. Native EndoFit Ovalbumin (InvivoGen) was used for all in vitro and in vivo experiments as a non-immunogenic control; though OVA-2pAMF is not expected to induce an immune response, such controls could prevent aberrant activation in control groups from non-specific immunostimulatory activity of OVA-2pAMF. We then used the RAW-Blue NF-κB reporter cells to test downstream signaling of the TLR9 receptor. Again, signaling induced by OVA-CpG was increased relative to unlinked controls (Fig. 3B). These results validated that conjugation of CpG to OVA-2pAMF enhanced TLR9 signaling. TLR9 is expressed in the endocytic compartment; thus, the divalency of the OVA-CpG construct both facilitates enhanced endosomal uptake through conjugation and increases TLR activation in the endosome to facilitate activation of APCs. Indeed, linked antigen-adjuvant formulations have been shown previously to enhance APC activation in vitro^15^.

After confirming activity of our conjugates, we tested their capability relative to unlinked controls to induce cross-presentation and activate dendritic cells in vitro*.* The DC2.4 dendritic cell line was used as APCs, and cross-presentation was quantified by co-culturing with B3Z hybridoma T-cells. These T-cells specifically recognize the OVA MHC-I epitope motif, SIINFEKL, presented on H-2 Kb. Upon recognition, β-galactosidase is produced which is quantified using a colorimetric substrate, CPRG. At concentrations ≥ 10 μg/mL, linked OVA-CpG incubated with the DC2.4 cells for 5 h invoked greater cross-presentation than unlinked controls (Fig. 3C). Indeed, the unlinked OVA + CpG was unable to induce cross-presentation even at the highest dose tested; we believe that this unexpected result might occur due to changes in the kinetics of OVA-CpG uptake relative to soluble CpG at the 5 h timepoint as well as enhanced TLR9 binding resulting from co-localization of OVA and CpG in a single endosome. These results highlight the importance of enhanced uptake and high endosomal adjuvant concentrations in facilitating cross-presentation.

To further demonstrate that TLR9 signaling is at the basis of the enhanced CD8^+^ T cell activation, we then evaluated dendritic cell activation resulting from treatment with our conjugates. CD80 and CD86 are common cell surface markers which would serve as a viable proxy of activation. OVA-CpG or unlinked controls were incubated with DC2.4 cells at 50 μg/mL for 20 h, and cells were subsequently stained and analyzed using flow cytometry. The DC2.4 cells showed enhanced cell surface expression of CD80, but not CD86, when treated with OVA-CpG relative to unlinked controls (Figs. 3D,E and S7). Previous studies have shown that both mature and immature DC2.4 cells highly express CD86, but not CD80, which could explain differences in their expression patterns in this model^23^. Moreover, a distinct activated DC population marked by an increase in granularity was observed by flow cytometry after treating the cells with linked conjugates relative to their unlinked controls, further validating the activated phenotype (Figs. 3F and S8). Higher cross-presentation and activation efficiency at lower CpG concentration demonstrates effectiveness of our conjugation strategy.

### In vivo vaccination experiments

After characterizing our conjugates in vitro, we designed an in vivo experiment to assay cellular and humoral immune responses to the conjugate OVA-CpG systems. Previous experience in our laboratory indicated that 10–50 μg of OVA and 25–50 μg of CpG are optimal for murine vaccination; however, given the linked nature of our conjugates, these doses were infeasible. We therefore implemented a dose of 1 nmol of the OVA-CpG conjugate (44 μg OVA + 12 μg CpG for a loading of 1.5 CpG/OVA) and corresponding amount of unlinked controls (44 μg EndoFit Ovalbumin + 12 μg uncapped CpG-SH). 1 nmol of unadjuvanted OVA and PBS were used as controls. A boost was administered 14 days after initial intramuscular injection, and humoral and cellular immune responses were assayed after 21 and 28 days, respectively.

In response to the vaccination schedule (described in Fig. S9A), no changes in body weight were observed, although OVA-CpG conjugates induced increased IFN-γ and MCP-1 production 24 h after injection (Fig. S9). This could result from greater TLR9 stimulation in the draining lymph nodes facilitated by conjugation to OVA, as observed with other nanoparticulate constructs^5^. After 21 days, no differences in total or IgG1/IgG2c antibody titers were observed between the linked and unlinked groups (Figs. S10,S11), indicating that the conjugates did not improve humoral immunity or induce Th1/Th2 bias in the immune response. The ability of antigen-adjuvant conjugates to enhance antibody production is disputed in the literature^9,11,16^. Our result suggests that OVA-TLR9 agonist linkage enhances the efficiency of endosomal uptake and TLR activation rather than global antigen recognition, providing little advantage for humoral immunity. After 28 days, spleens were harvested, cultured, and stained using antigen-specific MHC tetramers to assay the cellular response. The conjugates showed > 3 × enhancement in antigen-specific T-cell production (Fig. S11) indicating enhanced in vivo cellular immune responses.

**Figure 4:**
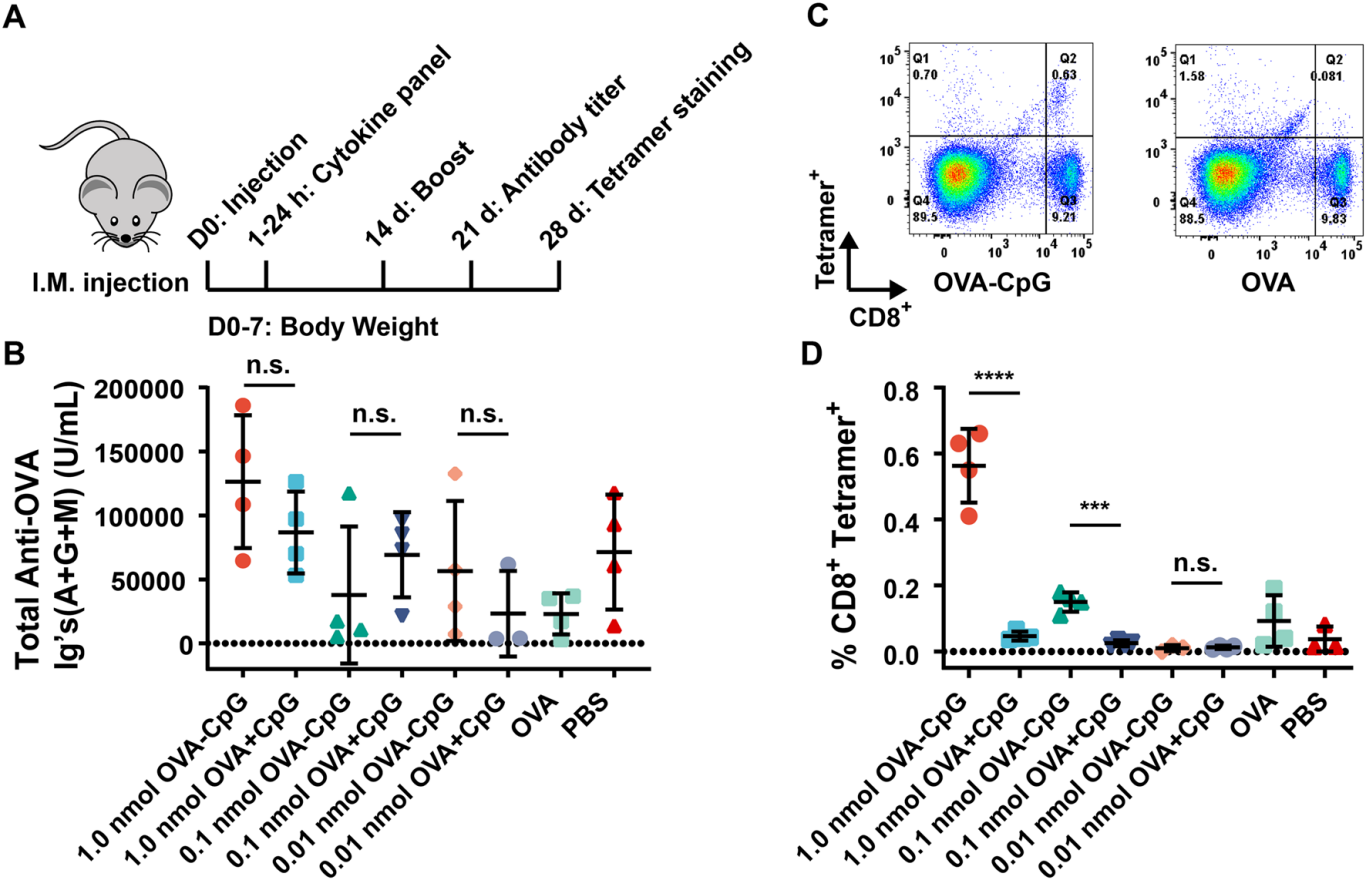
(**A**) Second in vivo experiment overview. (**B**) No significant differences in anti-OVA total antibody titers were observed 21 days after injection (p > 0.05). (**C**) Representative flow cytometry gating for evaluation of antigen specific CD8^+^ T-cell production. (**D**) Summary statistics for all flow cytometry gated as shown in (**C**) demonstrating enhanced antigen specific CD8^+^ T-cell production of OVA-CpG conjugates relative to unlinked controls. Statistics were conducted using student’s t-test to evaluate OVA-CpG relative to OVA + CpG.

Given these results, we reduced the dose of our linked vaccine system, running additional experiments at 1.0, 0.1, and 0.01 nmol (10 μg, 1.0 μg and 0.1 μg CpG for a loading of 1.2 CpG/OVA) using batch 2 of the OVA-CpG conjugates (as well as unlinked controls as described above) to observe the efficiency of our linked systems in generating cellular immunity (Fig. 4A). Again, serum anti-OVA antibody titers showed no differences after 21 days (Fig. 4B), while splenic antigen-specific CD8^+^ T-cell production was enhanced relative to unlinked controls at the 1.0 nmol dose. Intriguingly, a modest antigen-specific CD8^+^ T-cell response was also observed using the reduced 0.1 nmol OVA-CpG amount, which is less than one tenth of the CpG commonly used as an adjuvant. In contrast, minimal antigen-specific CD8^+^ T-cell production was observed using 1.0 or 0.1 nmol of unlinked control (Fig. 4C,D). Based on these results, we conclude CFPS is a scalable platform for generating antigens with site-specific nnAAs for conjugation to adjuvants. Our OVA-CpG conjugates reduced the quantity of adjuvant needed to invoke antigen-specific CD8^+^ T-cell production in our vaccine model.

## Conclusion

We demonstrate herein that CFPS is a facile technique to synthesize large quantities of protein containing site-specific nnAAs for antigen-adjuvant conjugation without disrupting protein folding or epitope recognition. Our OVA-CpG conjugates enhanced cross-presentation compared to unlinked controls in vitro, corresponding to increased antigen-specific CD8^+^ T-cell production in vivo. Moreover, our OVA-CpG conjugates reduced the dose of CpG needed to invoke antigen-specific CD8^+^ T-cell production tenfold. Our results suggest that CFPS can be used as a reliable method to produce antigenic proteins with efficient, site-specific conjugation of adjuvants. Given the need to generate potent CD8^+^ T-cell responses in both prophylactic and protective vaccines, future work using the antigen-adjuvant conjugation strategy described herein will focus on translating this model system to clinically relevant vaccines against disease. In particular, the antigen-adjuvant conjugation strategy holds promise to enhance responses at reduced doses in vaccines protecting against viral infections, such as influenza, where CD8^+^ T-cell responses are critical but inflammation limits the use of synthetic TLR agonists at efficacious doses. This strategy also holds promise to improve robustness of protective responses in bacterial polysaccharide conjugate vaccines, such as those targeted against pneumococcal or streptococcal bacteria, which suffer from poor immunogenicity. This method can overcome the limitations of conventional antigen-adjuvant conjugation and will be another useful approach for the treatment and prevention of disease.

## Methods

### Mice and materials

All chemical reagents unless noted were obtained from Sigma Aldrich. 5′ThioMC6-capped CpG1018 was obtained from IDT Tech. DBCO-Maleimide (Catalog# A108) was obtained from Click Chemistry Tools. RAW-Blue NF-κB, HEK mTLR9 SEAP-reporter cells, and EndoFit Ovalbumin (Catalog# vac-pova) used for control assays were obtained from InvivoGen. DC2.4 cells were obtained from EMD Millipore. B3Z T-cell hybridomas were obtained as a gift from N. Shastri (UC Berkeley). All cell culture reagents were obtained from Thermo Fisher Scientific. Anti-CD16/32 (clone 2.4G2), FITC anti-CD86 (clone PO3), and APC anti-CD80 (clone 16-10A1) were obtained from BioLegend. DC2.4 cells were cultured in RPMI-1640 supplemented with 10% FBS, 10 mM HEPES, 100 µM non-essential amino acids and 50 µM β-mercaptoethanol. B3Z cells were cultured in RPMI-1640 supplemented with 10% FBS and 50 µM β-mercaptoethanol. RAW-Blue and HEK mTLR9 cells were cultured in DMEM supplemented with 10% FBS and selective antibiotics. Cells were maintained at 37 °C and 5% CO_2_. C57Bl/6J mice were obtained from Jackson Laboratories and acclimatized for 1 week prior to experimentation. All experiments were conducted with approval of the University of Chicago Institutional Animals Care and Use Committee and in accordance with ARRIVE guidelines, and animals were maintained in accordance with guidelines and regulations defined by the National Institutes of Health. All statistical analyses were performed using GraphPad Prism.

### Cloning, expression, and purification of OVA-2pAMF

The codon-optimized gene for the expression of OVA containing two non-native amino acid, *p*-azidomethyl phenylalanine (pAMF) sites, K20pAMF and K370pAMF, was synthesized at ATUM (Menlo Park, CA) and subcloned with an N-terminal methionine into a proprietary vector. The final gene for OVA-[K20pAMF/K370pAMF]-TEV-his_6_ contains a C-terminal TEV protease site (ENLYFQG) followed by a his_6_-affinity tag for purification. In vitro protein expression using cell free protein synthesis was performed as described elsewhere^19^. For titer estimates, expression of native or pAMF-containing OVA genes was monitored by incorporation of ^14^C-leucine (GE Life Sciences, Piscataway, NJ). Autoradiography (Storm 840 PhosphoImager) was used to estimate total and soluble fractions of each protein. For large scale expression of OVA [K20/K370-pAMF]-TEV-his_6_, the DASbox Mini Bioreactor System (Eppendorf AG) was used. Expression was performed at 25 °C and pH 7.2 for 10 h with stirring at 650 rpm while sparging 30% oxygen in air through the reaction. After 10 h, the reaction mixtures were ultracentrifugated at 15,000 G at 4 °C for 30 min and filtered using a 0.45 µm filter. The crude filtrate was loaded onto a 5 ml HisTrap column and equilibrated in 10 mM imidazole in Buffer A (50 mM Tris, 10% Glycerol, 150 mM NaCl). The protein was eluted using a step gradient of 10–500 mM imidazole in Buffer A. The eluent was pooled, concentrated, and incubated with his_6_-tagged TEV protease overnight under dialysis against Buffer A. The dialyzed cleavage reaction was loaded onto a second pre-equilibrated, 5 ml HisTrap column and untagged OVA-2pAMF was collected. The crude OVA-2pAMF was concentrated and purified on a size exclusion column (Superdex 200 26/60 and Superdex 75 26/60 columns connected in tandem) pre-equilibrated with Buffer A. Finally, OVA-2pAMF containing fractions were pooled, 3 × diluted in Buffer B (50 mM Tris, 10% Glycerol, pH 8.0), and loaded onto Capto Q ImpRes anion exchange column pre-equilibrated with Buffer B. The bound protein was eluted using a linear gradient of 0–1 M NaCl in Buffer B. The eluent containing purified OVA-2pAMF was pooled and frozen at − 80 °C for further use.

### Synthesis of CpG-DBCO

To remove the 5′ thiol cap, MC6-capped CpG1018 (CpG-S–S-(CH_2_)_6_–OH) was shaken at 1500 rpm overnight with 100 mM tris(2-carboxyethyl)phosphine hydrochloride (TCEP-HCl) in PBS adjusted to pH 8.5 with 1 M NaOH. The oligonucleotide product was precipitated with ethanol, resuspended in PBS, and characterized by ESI–MS. Positive mode analysis of oligonucleotide conjugates was performed on a Waters Xevo G2 XS Q-TOF mass analyzer. A 5 min run in 50 mM pH 7.4 ammonium acetate was used to elute each sample off a Waters BEH 200 Å 150 mm SEC stationary phase at 0.1 mL/min. The concentration of DNA was verified by UV–VIS spectroscopy, and presence of thiol was confirmed by Ellman’s assay. A cyclooctyne handle was then introduced using DBCO-Maleimide to react with the free thiol. DBCO-Maleimide was dissolved at 10 mg/mL in DMSO, and 10 eq of this stock solution was added to 1 mL of CpG-SH in PBS. The reaction mixture was shaken at 25 °C overnight. Unreacted DBCO-Maleimide and trace DMSO was removed by passing the crude thrice through a 3 k MWCO Amicon centrifugal filter, and the product was characterized by ESI–MS. Complete reaction of the thiol was confirmed by Ellman’s assay, and concentration of DNA in the product solution was verified by UV–VIS spectroscopy on a Nanodrop 2000 instrument. CpG-DBCO was stored in PBS (1 mg/mL) at − 20 °C for later use.

### OVA-CpG synthesis

The OVA-CpG conjugate was prepared using azide-alkyne click chemistry. To a 1 mg/mL solution of OVA-2pAMF was added 2.5 eq CpG-DBCO (from the 1 mg/mL stock) at 35 °C for 48 h. Unreacted CpG-DBCO was removed by passing the crude thrice through a 30 k MWCO Amicon centrifugal filter. The purified product was characterized by SDS-PAGE gel electrophoresis. Samples were treated with 2.5% β-mercaptoethanol, heated to 90 °C, and separated by SDS-PAGE gel. Gels were stained with One-Step Blue Protein Gel Stain (Biotium), and imaged with an Azure c600 Imager (Azure Biosystems). Reaction extent was determined using ImageJ.

### RAW-blue assay

RAW-Blue cells were passaged and plated in a 96 well plate at 50,000 cells/well in 180 μL DMEM containing 10% HI-FBS and selective antibiotics. The cells were stimulated with the conjugates and unlinked controls for 20 h at 37 °C and 5% CO_2_. NF-κB activity was measured by a QUANTI-Blue (Invivogen) assay and the absorbance was measured at 620 nm using a Multiskan FC plate reader (Thermo Scientific).

### HEK mTLR9 assay

HEK mTLR9 cells were passaged and plated in a 96 well plate at 100,000 cells/well in 180 μL DMEM containing 10% HI-FBS and selective antibiotics. The cells were stimulated with the conjugates or unlinked controls for 20 h at 37 °C and 5% CO_2_. TLR9 binding was measured by a QUANTI-Blue (Invivogen) assay and the absorbance was measured at 620 nm using a Multiskan FC plate reader (Thermo Scientific).

### In-vitro cross presentation assay

The cross-presentation efficiency of conjugates was measured as described previously with minor changes^11^. DC2.4 cells (100,000 cells/well) were plated in 96-well plates and stimulated with linked OVA-CpG conjugates or unlinked controls for 5 h at 37 °C and 5% CO_2_. Subsequently, the media was replaced and DC2.4 cells were co-cultured with B3Z T-cell hybridomas (100,000 cells/well) for 18 h. The cells were centrifugated at 500 G for 5 min and the supernatant was removed. The cells were washed twice with 100 µL PBS. 0.15 mM CPRG reagent was prepared in lysis buffer (0.5% (v/v) NP-40 in PBS), 100 µL of the prepared reagent was added to each well, and cells were incubated at 37 °C for 12–16 h in the dark. β-gal activity was quantified by measuring absorbance at 570 nm using a Multiskan FC plate reader (Thermo Scientific).

### In vitro DC activation assay

DC2.4s (2 million cells/well) were incubated in untreated 24-well plates and treated with OVA-CpG conjugates or unlinked controls in 0.5 mL culture media for 20 h at 37 °C and 5% CO_2_. The cells were mechanically released from the plate and centrifuged at 2500 RPM at 4 °C for 10 min. The cell pellet was resuspended in 100 µL cold FACS buffer (10% FBS + 0.1% NaN_3_ in PBS) and incubated with anti-CD16/32 (Fc receptor blocking antibody) (1.0 μg/million cells) on ice for 10 min. The cell suspension was pelleted and the supernatant was removed. The cell pellet was then resuspended in cold FACS buffer and incubated with FITC anti-CD86 (1.0 μg/million cells) and APC anti-CD80 (0.5 μg/million cells) on ice in darkness for 30 min. Cells were washed twice with 300 μL cold FACS buffer, and then resuspended in FACS buffer (150 μL) and kept on ice until analysis. DC activation was assayed using an ACEA Novocyte flow cytometer and data were processed using the NovoExpress software.

### In vivo characterization

Mice were injected intramuscularly in each flank with 50 μL OVA-CpG conjugates, OVA + CpG, OVA, or PBS (n = 4/group). Body weight was measured prior to injection and daily for one week. Mice were bled via facial vein 1 and 24 h after injection, and serum cytokine production was assayed using LEGENDPlex Mouse Inflammation Panel 13plex (BD Biosciences). After 14 days, the same formulation was administered as a boost. After 21 days, serum was collected and antibody production was assayed using mouse Anti-Ovalbumin Ig's total (A + G + M) (Alpha Diagnostic International), Anti-Ovalbumin IgG1 (Alpha Diagnostic International), and Anti-Ovalbumin IgG2c (Chondrex) ELISA kits. After 28 days, mice were sacrificed and spleens were collected for tetramer staining. Spleens were homogenized, and cells were filtered through a 70 μm strainer. Red blood cells were lysed by incubating with ACK Lysing Buffer for 5 min at 25 °C. Splenocytes (2 million cells) were plated in a 96-well plate and incubated with anti-CD16/32 (Fc receptor blocking antibody) (1.0 μg/million cells) for 15 min at 4 °C. Splenocytes were washed with FACS buffer (PBS with 2% FBS), resuspended, and stained with APC MHC Class I tetramers (Tetramer Shop) at 37 °C in the dark. After 15 min, FITC anti-CD8 (BD Biosciences) was added and incubated for 30 min longer. Splenocytes were washed and resuspended in FACS buffer. Antigen-specific T-cell production was assayed using an ACEA Novocyte flow cytometer, and data were processed using FlowJo.

## Supplementary Information

Supplementary Information
