# Supplementary material for: Site-specific antigen-adjuvant conjugation using cell-free protein synthesis enhances antigen presentation and CD8+ T-cell response
Source: Sci Rep. 2021 Mar 18;11:6267. doi: 10.1038/s41598-021-85709-1 (PMC7973483; doi:10.1038/s41598-021-85709-1)
Supplement: Supplementary file 1 — Supplementary Information [file 41598_2021_85709_MOESM1_ESM.docx]

**Supporting information**

**Site-specific antigen-adjuvant conjugation using cell-free protein synthesis enhances antigen presentation and CD8^+^ T cell response.**

Adam M. Weiss^1,2,*^, Jainu Ajit^1,*^, Tyler Albin^1,3,*^, Neeraj Kapoor^4^, Shilpa Maroju^4^, Aym Berges^4^, Lucy Pill^4^, Jeff Fairman^4,^^, Aaron P. Esser-Kahn^1,#^

^1^ Pritzker School of Molecular Engineering

University of Chicago

5640 S. Ellis Ave., Chicago, IL 60637, USA

^2^ Department of Chemistry

University of Chicago

5735 S Ellis Ave., Chicago, IL 60637, United States

^3^ Department of Chemistry

University of California, Irvine

1102 Natural Sciences 2, California 92617, United States

^4^ Vaxcyte, Inc.

353 Hatch Drive, Foster City, California 94404, United States

* These authors contributed equally

^^^ E-mail: [jfairman@sutrovax.com](mailto:jfairman@sutrovax.com)

^#^ E-mail: [aesserkahn@uchicago.edu](mailto:aesserkahn@uchicago.edu)

**S1. Experimental Methods**

*Multi-Angle Light Scattering (MALS) Analysis*

The SEC MALS UV-RI setup consists of an Agilent HPLC 1100 degasser, temperature-controlled auto-sampler (4°C), column compartment (25°C), and UV-VIS diode array detector (Agilent, Santa Clara, CA) in line with a DAWN-HELEOS multi-angle laser light scattering detector and Optilab T-rEX differential refractive interferometer (Wyatt Technology, Santa Barbara, CA) coupled to three TOSOH columns in series: TSKgel Guard PWXL 6.0 mm ID x 4.0 cm long, 12 µm particle;  TOSOH TSKgel 6000 PWXL 7.8 mm ID x 30 cm long, 13 µm particle; and a TSKgel 3000 PWXL 7.8 mm ID x 30 cm long, 7µm particle. A mobile phase consisting of 0.2 µm filtered PBS was used at a 0.5 mL/min flow rate and 50-100 µg sample was injected for analysis. Agilent Open Lab software was used to control the HPLC, and Wyatt Astra 7 software was used for data collection and molecular weight analysis.

*pAMF site confirmation using DBCO-TAMRA labeling*

Purified OVA-2pAMF (50 µM) was incubated with excess of Dibenzocyclooctyne-PEG4-tetramethylrhodamine (DBCO-TAMRA) dye (5 mM) for 1 h to label azide moieties. Thereafter, the reactions were analyzed using SDS-PAGE gel and fluorescence readout was recorded using a Syngene G-box gel imager.

*Mass spectrometry of OVA-2pAMF*

Positive mode analysis of proteins was performed on a Waters Xevo G2 XS Q-TOF mass analyzer. A 5 min gradient from 0% B to 97% B was used to elute the protein off a Waters BEH phenyl 300 Å stationary phase at 0.2 ml/min. Mobile buffers were prepared gravimetrically; phase A consisted of 0.1% formic acid in water while phase B was 0.1% formic acid in acetonitrile. Waters Masslynx MaxEnt1 software was used to deconvolute the charge-state ladder into a deconvoluted mass.

*Endotoxin Removal*

Endotoxin removal was conducted as previously reported with minor alterations.^[S1]^ Briefly, a 5% Triton X-114 solution in PBS was cooled in an ice bath, and 100 μL of this solution was added to 400 μL protein solution. The solution was shaken for 40 min at 4°C, warmed to 37°C for 10 min, and ultracentrifugated at 37°C for 10 min at 12,000 G. The top layer containing protein was collected, and the lower layer discarded. This procedure was repeated three times, and protein solution was incubated with SM2 Bio-Beads (Bio Rad) overnight to remove residual Triton X-114. Endotoxin removal <1.5 EU/mL was validated by diluting 2 μL protein solution with 198 μL LAL Reagent Water and performing 0.015 EU/mL ToxinSensor Single Test Assay Kit (GenScript) according to the manufacturer’s procedure. Protein concentration before and after endotoxin removal was determined by Pierce BCA Assay Kit (Thermo Scientific).

*Size-exclusion HPLC*

Aggregate and sample purity analysis using size-exclusion HPLC was conducted on an Agilent 1260 Infinity system equipped with a Yarra 3 µm SEC-2000 300 x 4.6 mm LC Column. A 15 min isocratic elution in 100 mM pH 6.8 phosphate buffer was used to separate samples, and samples were monitored at 254 and 280 nm to resolve peaks.

*Anion Exchange Chromatography*

Separation of OVA-CpG components was conducted using a HiTrap Q FF 1 mL anion exchange column (Cytiva). Briefly, the column was flushed with 5 mL PBS (pH = 6.8). Then, the OVA-CpG mixture was loaded onto the column, and the column was washed with 5 mL PBS. A stepwise elution was subsequently conducted by eluting 5 mL of 0.3, 0.4, 0.5, and 1.0 M NaCl in PBS (pH = 6.8). The fractions were collected and concentrated using 30k MWCO Amicon centrifugal filters. The concentrated fractions were the characterized by SDS-PAGE gel electrophoresis. Fractions were treated with 2.5% β-mercaptoethanol, heated to 90°C, and separated by SDS-PAGE gel. Gels were stained with One-Step Blue Protein Gel Stain (Biotium), and imaged with an Azure c600 Imager (Azure Biosystems). Reaction extent was determined using ImageJ.

**S2. Supplementary Figures**


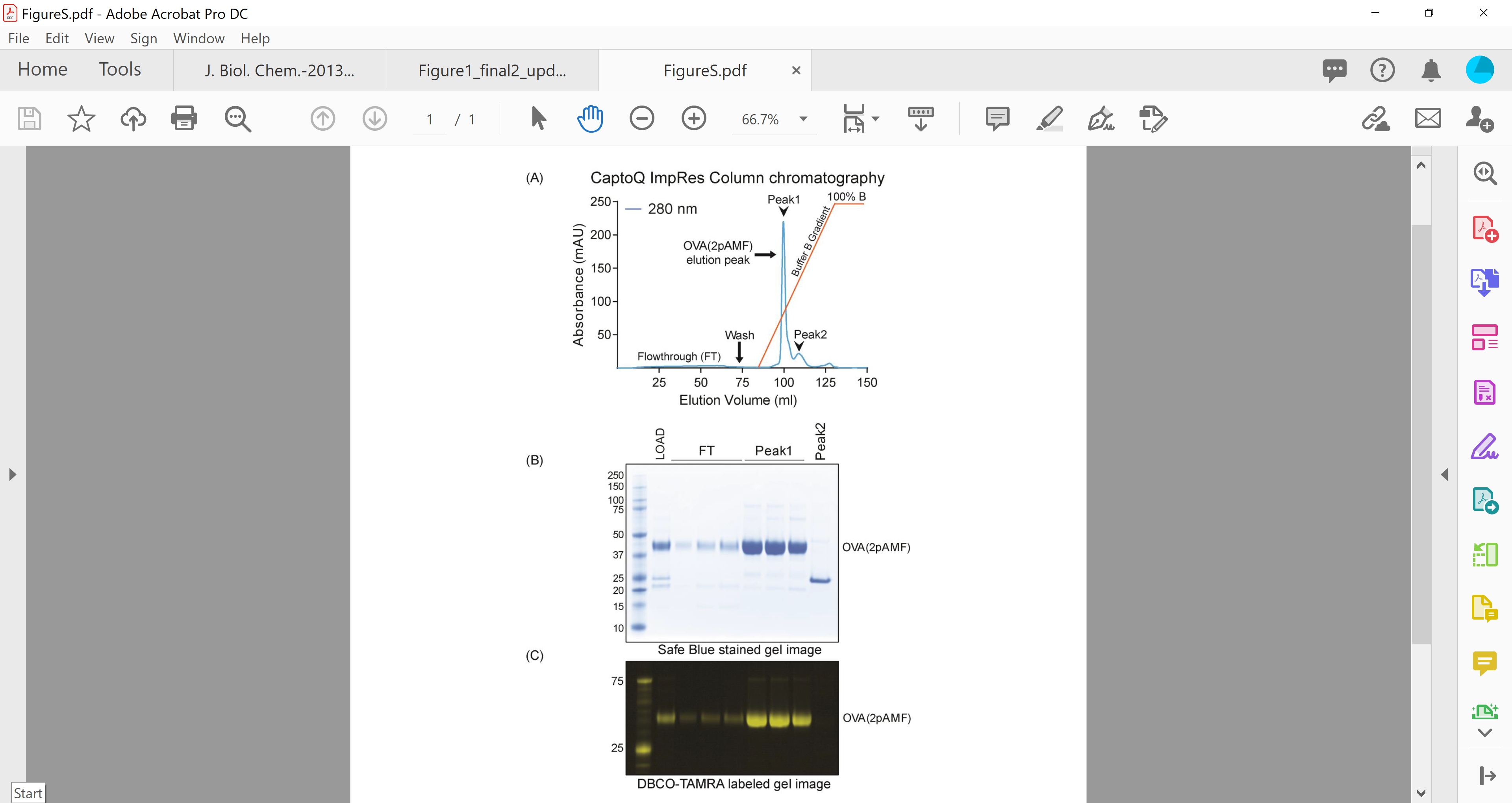


**Figure S1**: Purification of OVA-2pAMF. (A) Anion exchange chromatogram showing the final step of purification followed by (B) safe-blue stained and (C) DBCO-TAMRA labeled SDS-PAGE analysis of the FT and elution fractions.


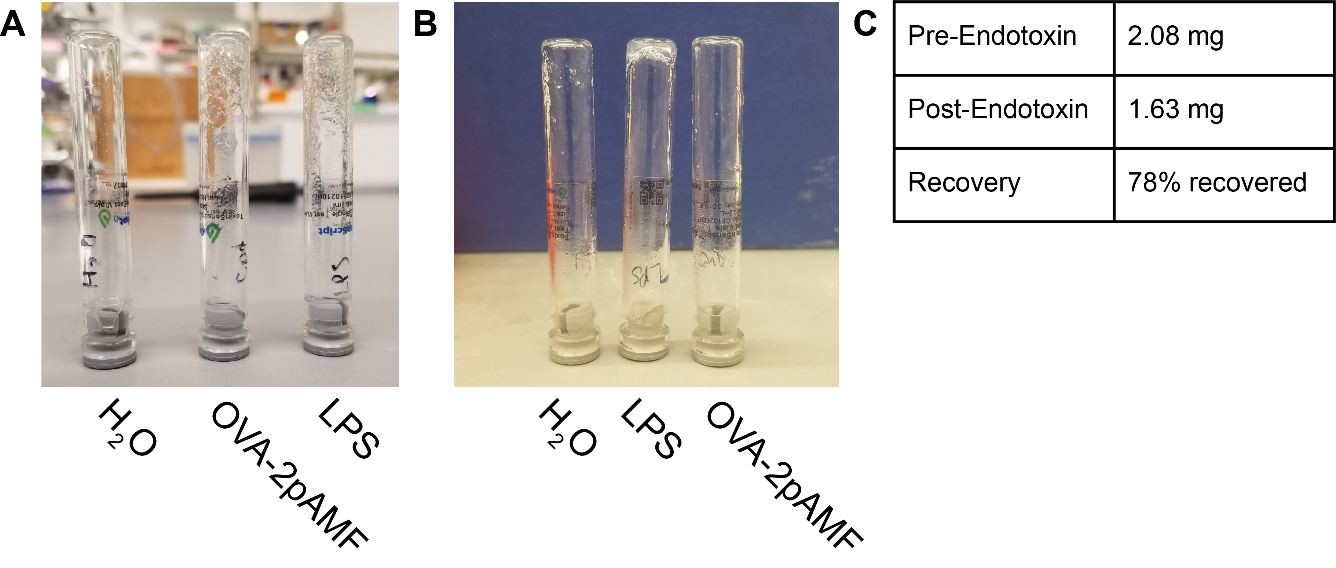


**Figure S2**: LAL Assay results A) before and B) after endotoxin was removed from OVA-2pAMF using three Triton X-114 washes. Absence of clotting indicates endotoxin decontamination < 1.5 EU/mL. C) BCA Assays conducted before and after endotoxin decontamination reveal 78% recovery after three washes.


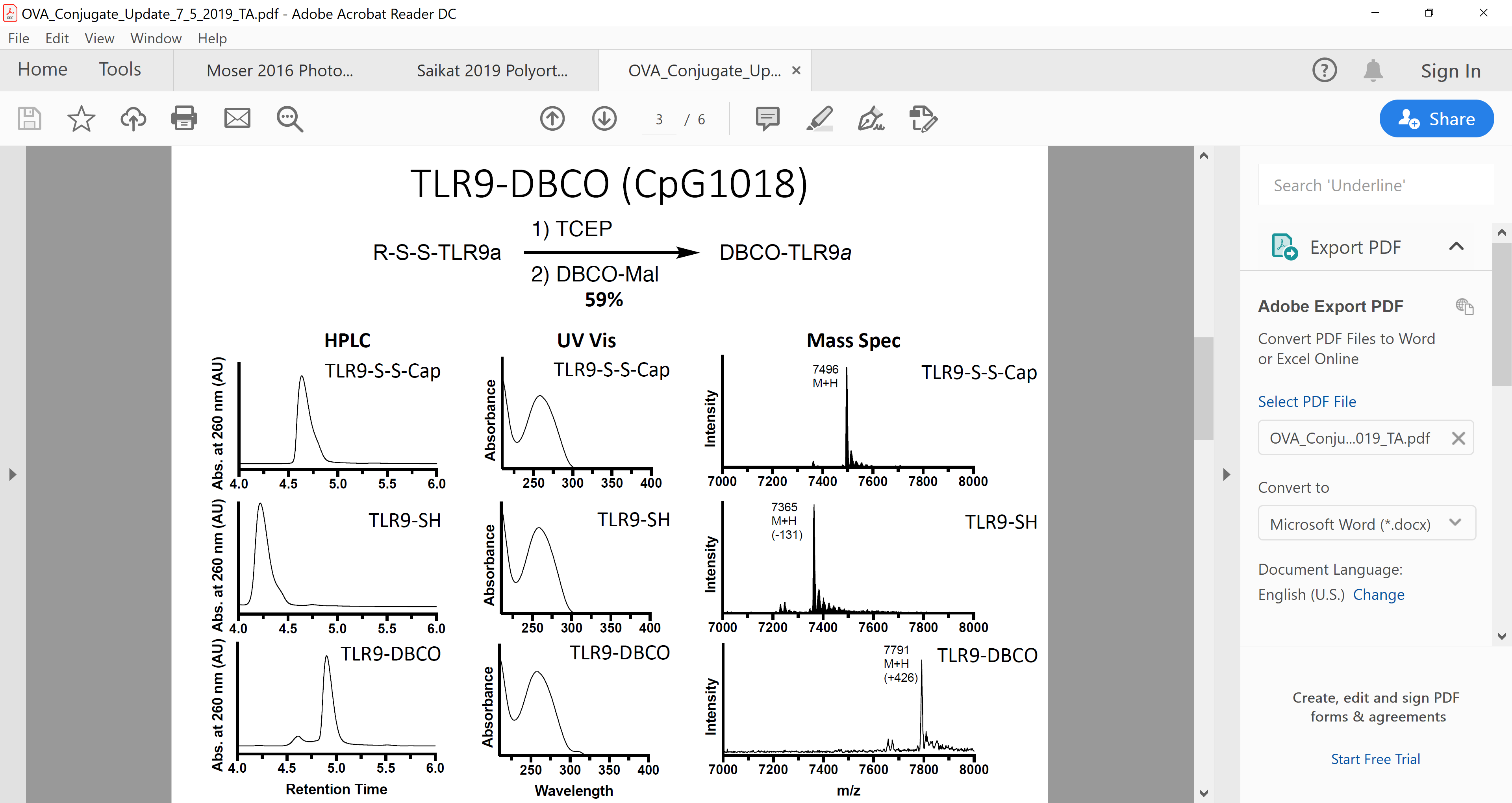


**Figure S3**: Functionalization and characterization of CpG. Capped CpG samples (R = -(CH_2_)_6_-OH) were treated first with TCEP to release the free thiol. The free thiol was subsequently reacted with DBCO-Maleimide to obtain an alkyne-modified derivative. Samples were characterized by HPLC, UV-VIS, and Q-TOF ESI-MS at each characterization step as shown to validate that the modifications were successful.


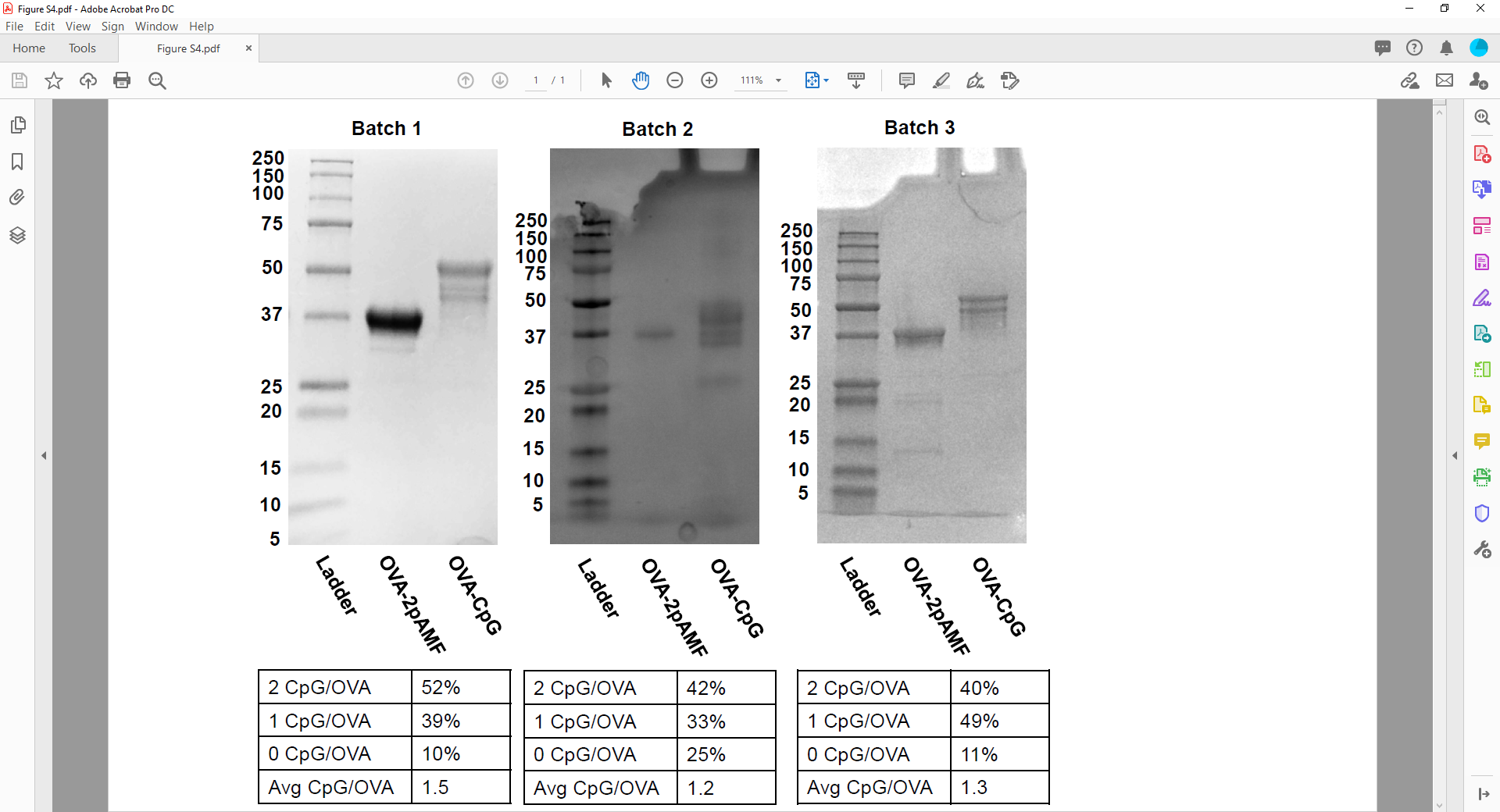


**Figure S4:** Full gels and densitometry of OVA-CpG conjugates. Gels were stained using One-Step Blue Protein Gel Stain, and densitometry was conducted using ImageJ to obtain the CpG content per OVA.


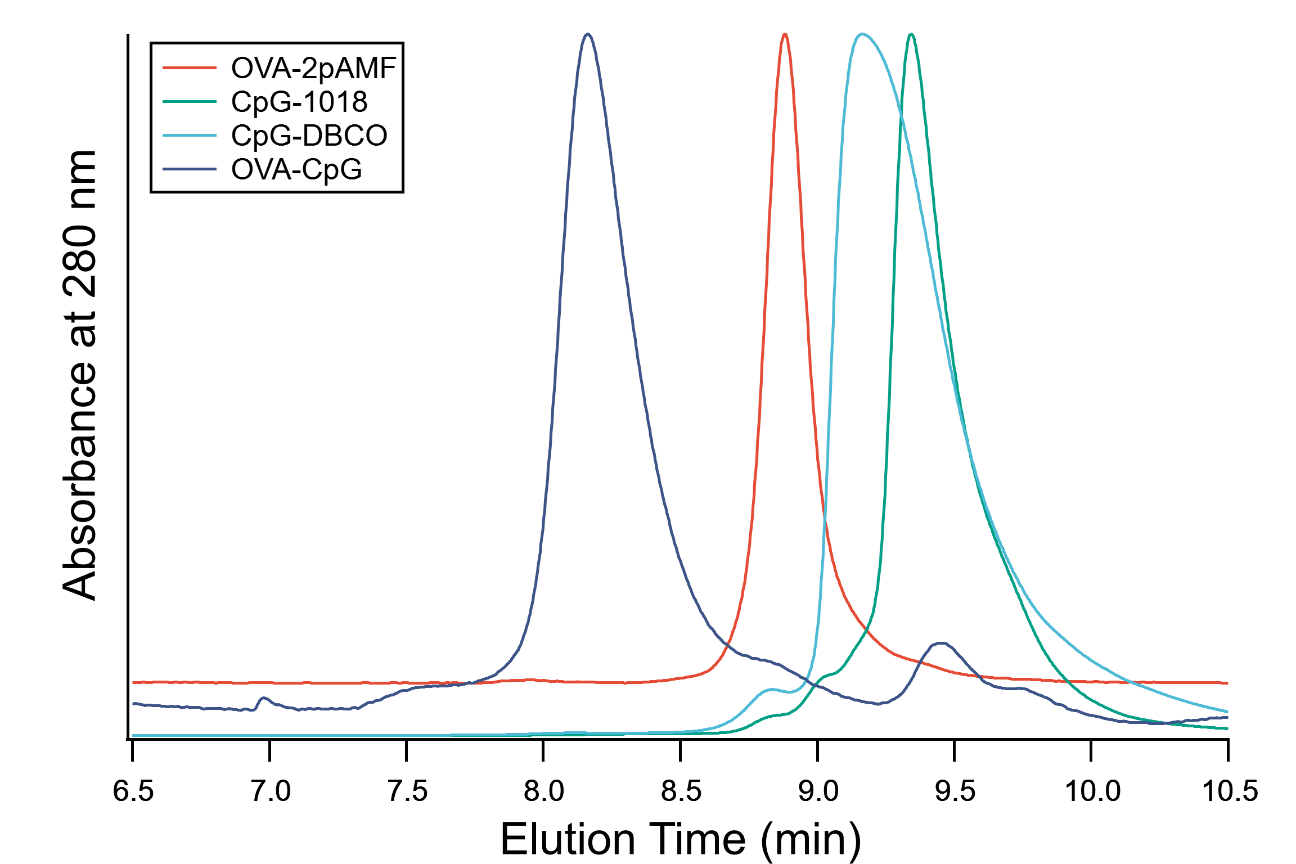


**Figure S5:** Size exclusion HPLC of OVA-CpG and component species reveals sufficient removal of CpG-DBCO and absence of high molecular weight aggregates in the OVA-CpG sample. Samples were eluted using 100 mM pH 6.8 phosphate buffer on a Yarra SEC-2000 300Å column and monitored at 280 nm. It should be noted that OVA-(CpG)_2_ and OVA-(CpG)_1_ elute as a single fraction at 8.1 min, which was verified by collecting the fractions and submitting them to SDS-PAGE gel chromatography (not shown).


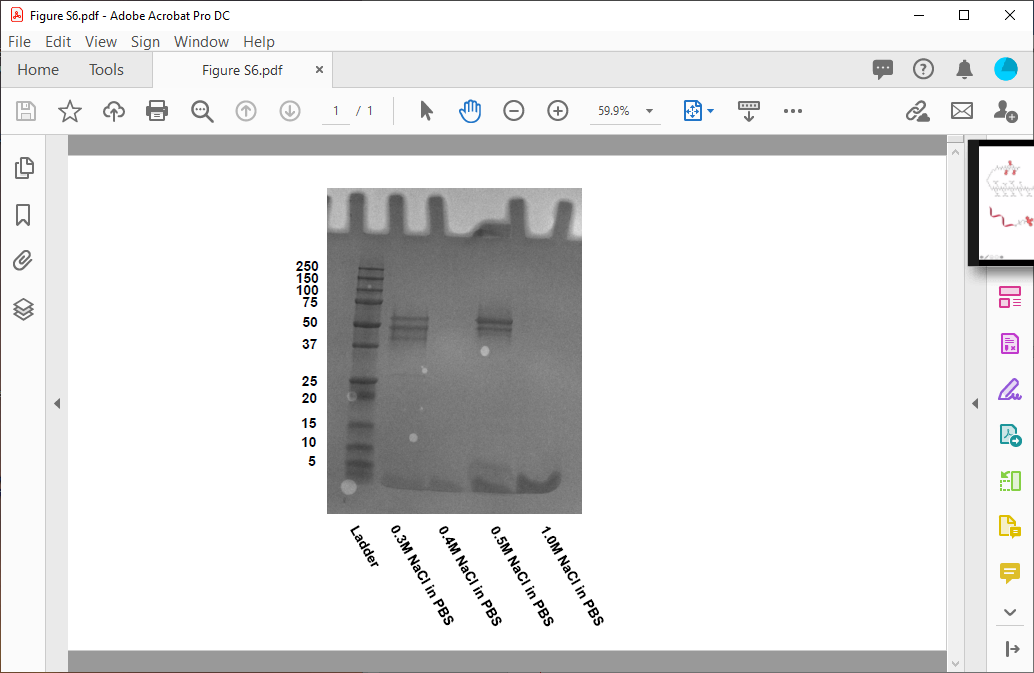


**Figure S6:** Anion exchange chromatography of OVA-CpG was attempted to isolate OVA-CpG fractions with different loading of CpG, and SDS-PAGE gel chromatography was used to indicate the purification of fractions containing 0, 1, or 2 CpG/OVA. A band containing 1+2 CpG/OVA eluted at a concentration of 0.5 M NaCl in PBS.


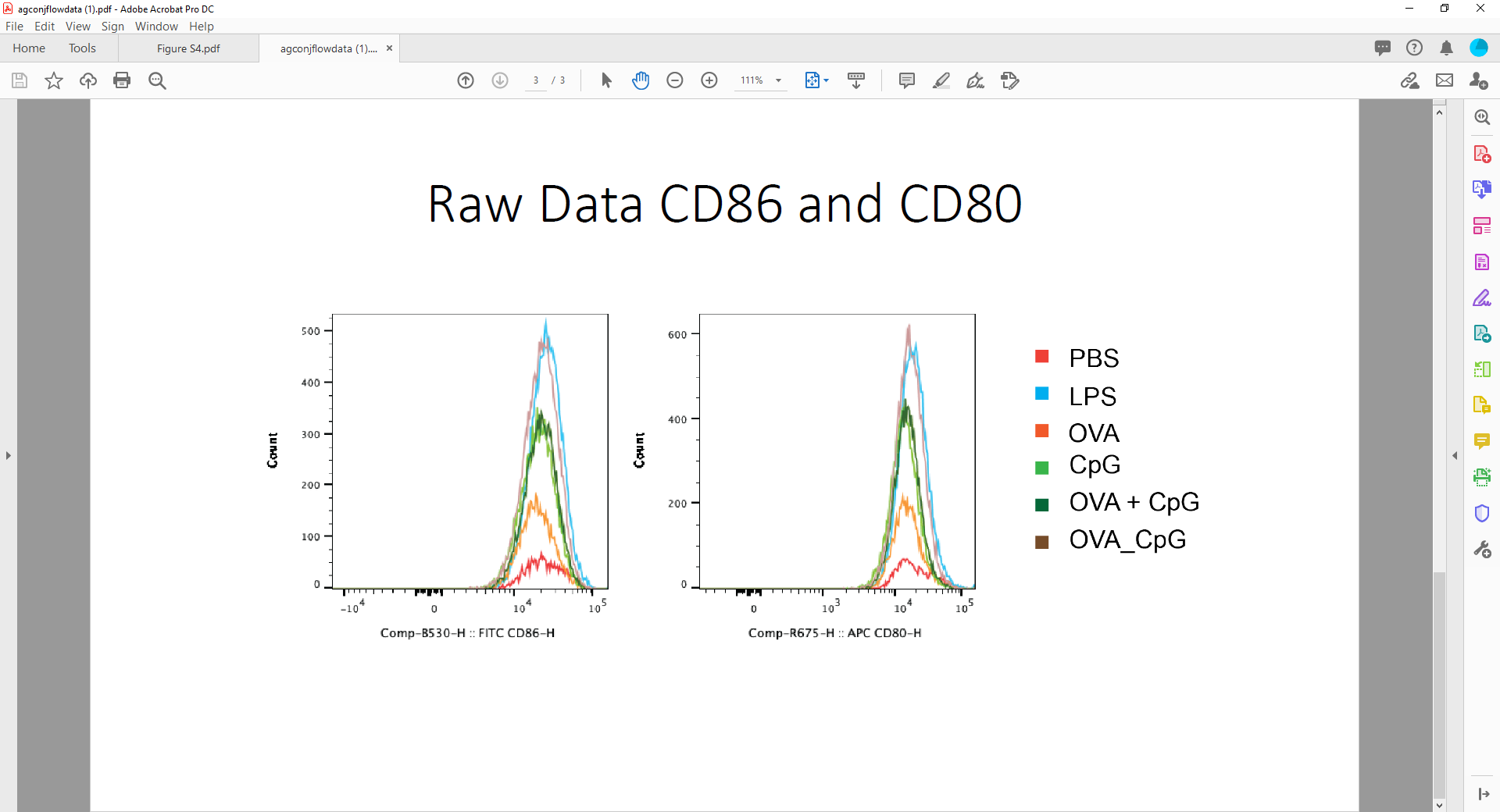


**Figure S7:** Fluorescent intensity plots of CD86 (left) and CD80 (right) expression after DC2.4 cells were incubated for 20 h with 50 μg/mL OVA-CpG or unlinked controls, stained, and analyzed using flow cytometry.


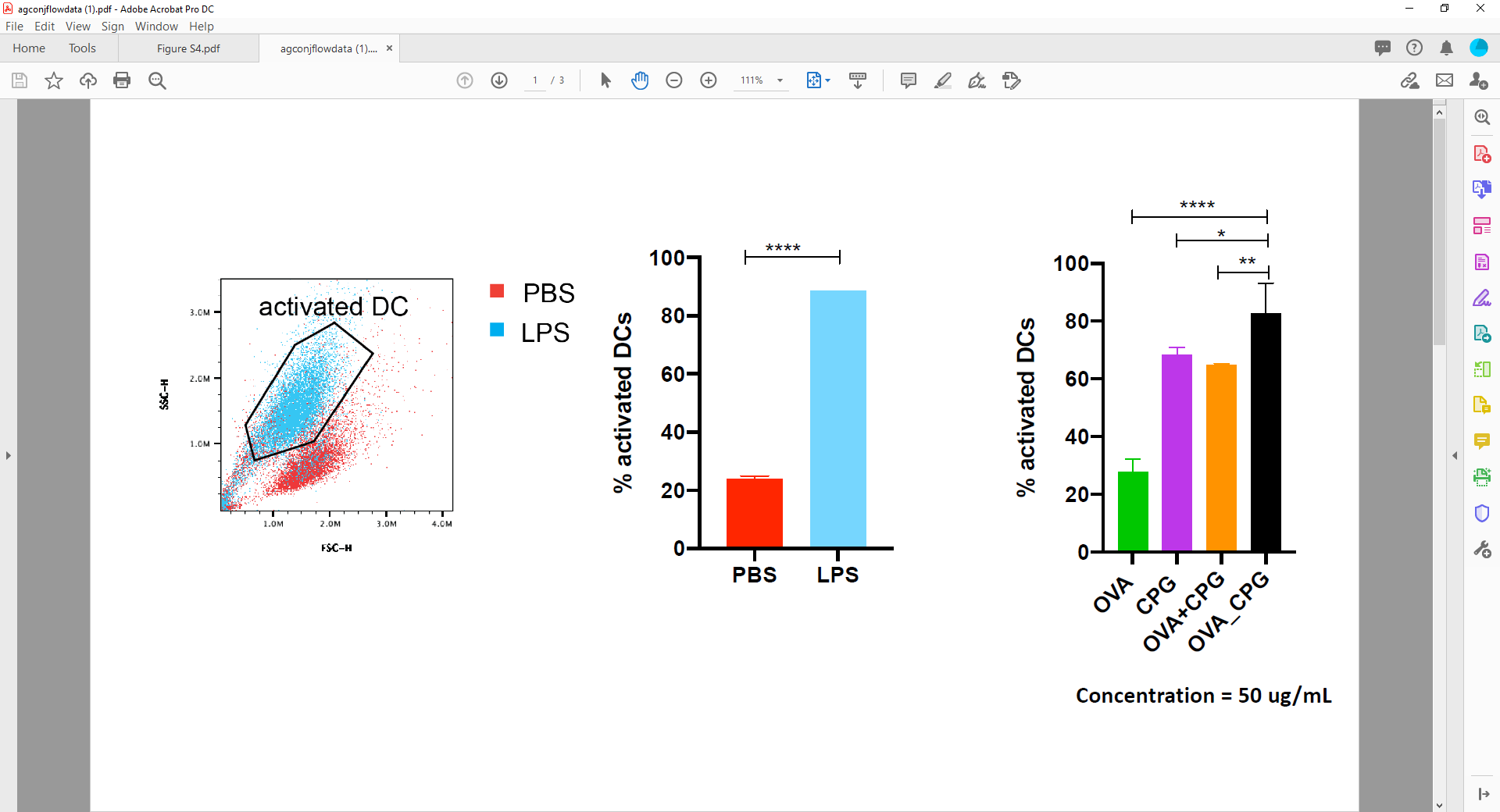


**Figure S8:** Plot of FSC-H against SSC-H showing gating used in Fig. 3F and demonstrating enhanced granularity in the activated DC2.4 cell population after treatment with 100 ng/mL LPS relative to PBS.


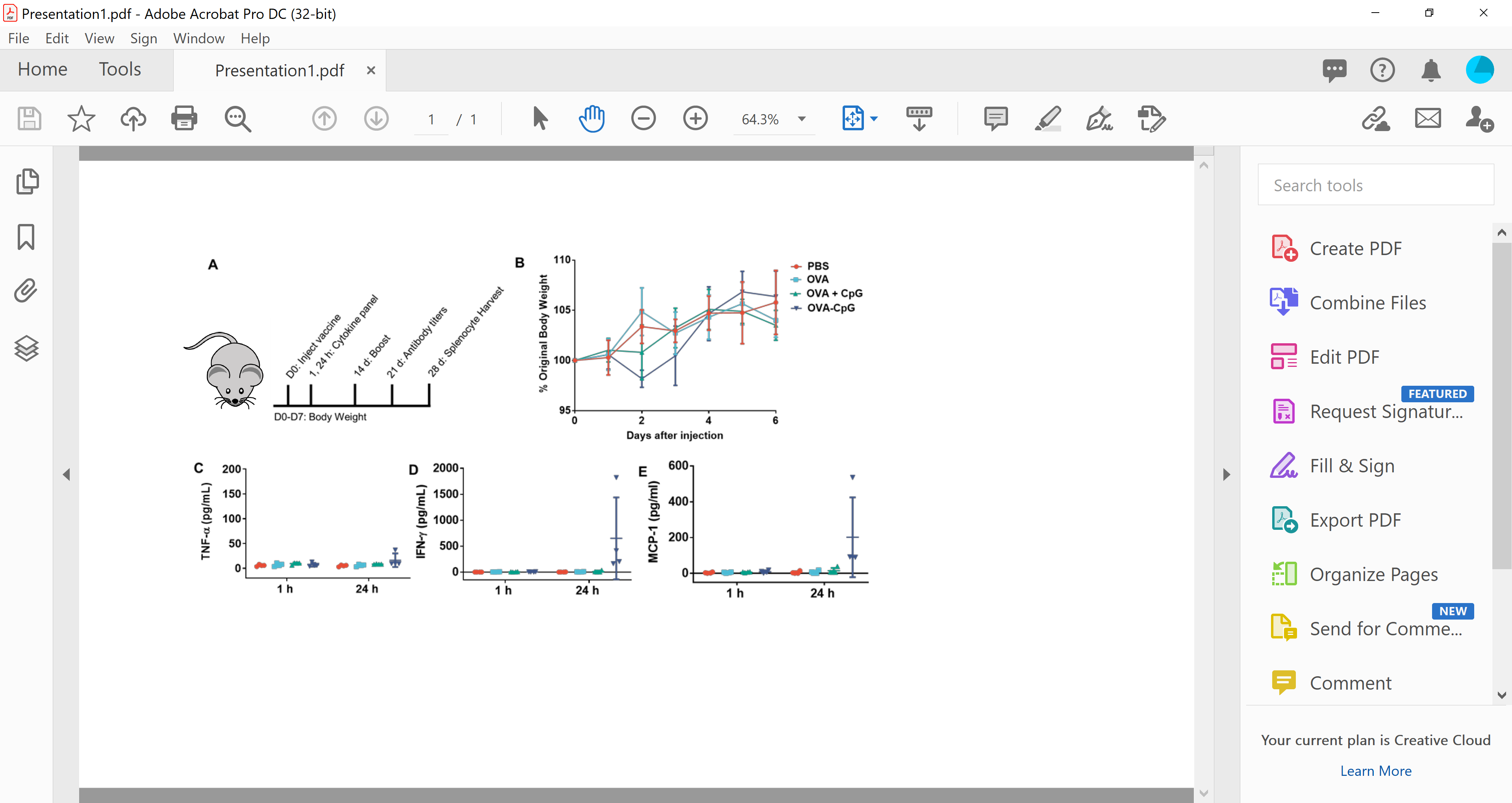


**Figure S9:** Acute inflammatory response to injection of OVA-CpG after the vaccination schedule for the first *in-vivo* experiment using 1 nmol of OVA-CpG (batch 1) or unlinked controls as defined in (A). B) No significant changes in body weight were observed after injection. C) No significant changes in TNF-α secretion was observed 1 or 24 h after injection. After 24 h, significant D) IFN-γ and E) MCP-1 production was observed.


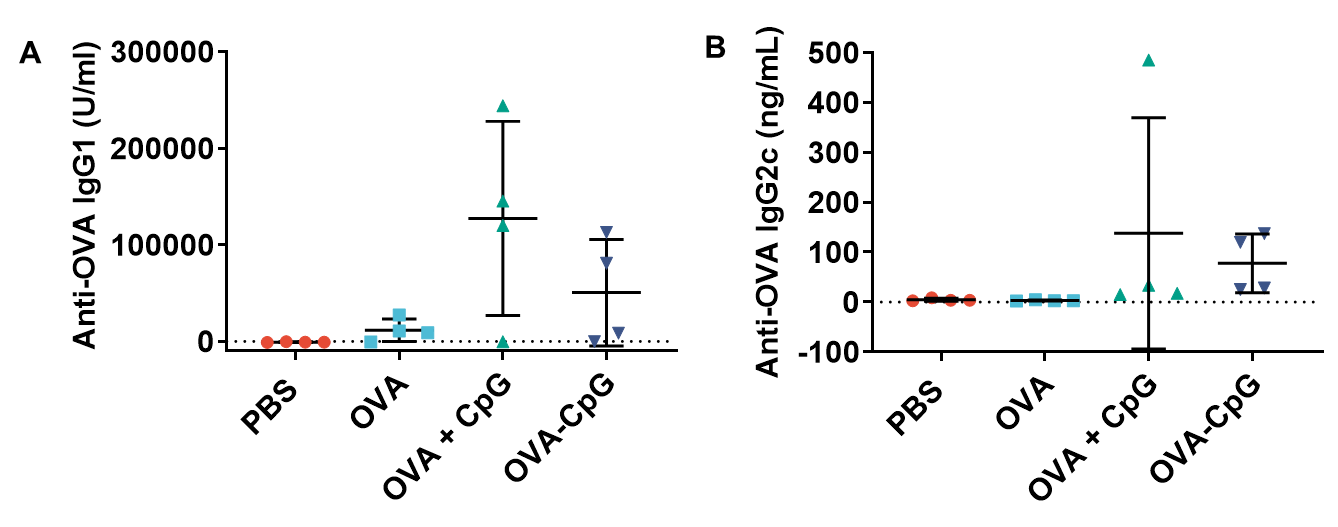


**Figure S10:** Mice were vaccinated according to the schedule in Fig. S6A, and serum was collected after 21 d for ELISAs. No differences in A) IgG1 or B) IgG2c specific anti-OVA titers were observed 21 d after injection.


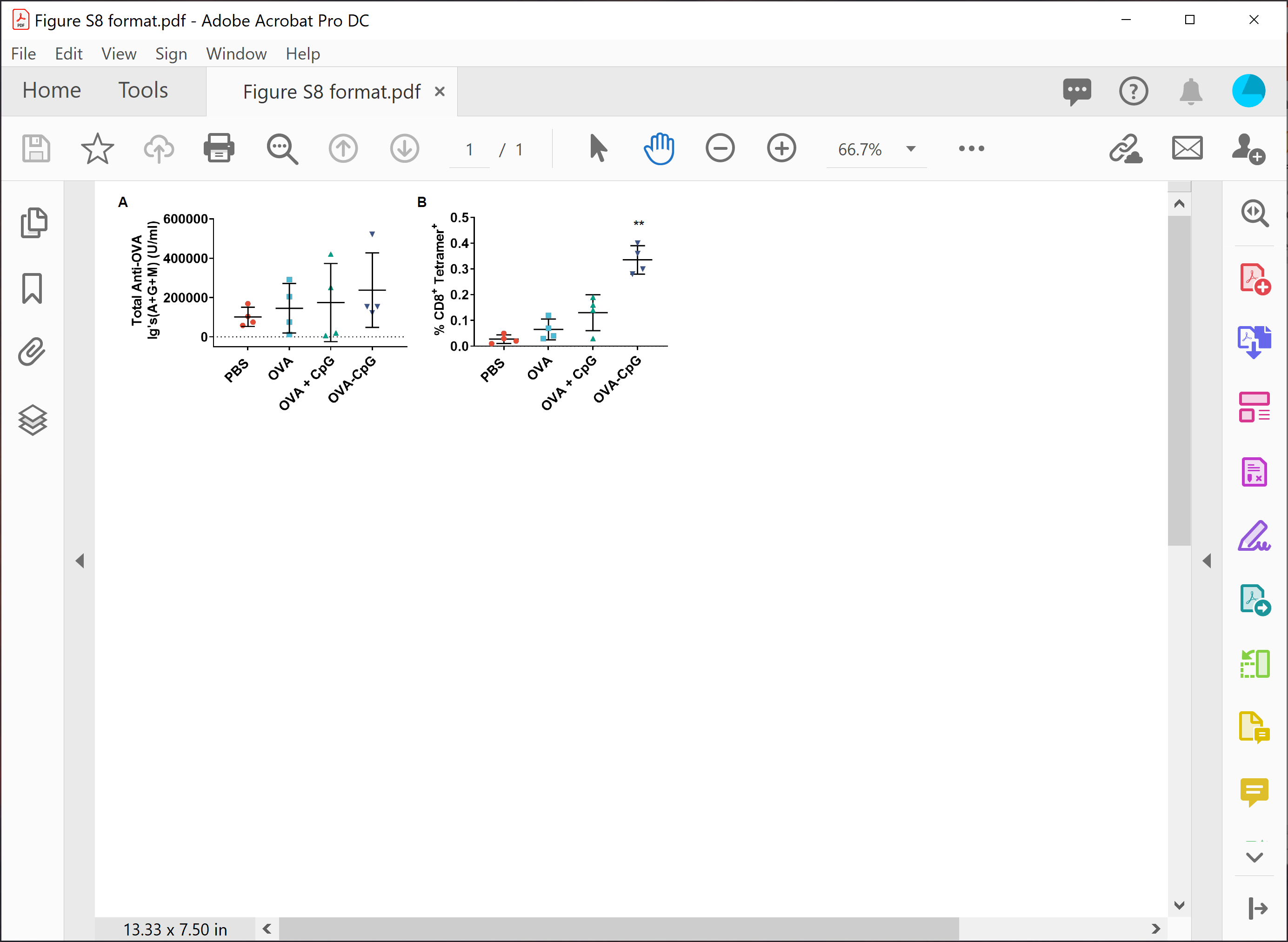


**Figure S11:** Mice were vaccinated according to the schedule in Fig. S6A, and serum was collected after 21 d for ELISAs. Mice were sacrificed after 28 d and T cells were harvested for tetramer staining. A) No differences in total anti-OVA Ig(G+A+M) titers were observed 21 d after injection. B) OVA-CpG induced significant increase (**, p < 0.01) relative to OVA + CpG in splenic antigen-specific T-cell production 21 d after injection. Statistics were conducted using student’s t-test to evaluate OVA-CpG relative to OVA + CpG.

**S3. Supplementary References**

[S1] Teodorowicz, M.; Perdijk, O.; Verhoek, I.; Govers, C.; Savelkoul, H. F.; Tang, Y.;

Wichers, H.; Broersen, K., Optimized Triton X-114 assisted lipopolysaccharide (LPS) removal method reveals the immunomodulatory effect of food proteins. *PLoS One* **2017**, 12 (3), e0173778.
